# Enhanced dynamic wedge output factors for Varian 2300CD and the case for a reference database

**DOI:** 10.1120/jacmp.v16i5.5498

**Published:** 2015-09-08

**Authors:** Christopher F. Njeh

**Affiliations:** ^1^ Department of Radiation Oncology The Diagnostic and Treatment Center, Marshfield Clinic Weston WI USA

**Keywords:** enhanced dynamic wedges, wedge factors, reference database, linear accelerator, Varian, output factors

## Abstract

Dose inhomogeneity in treatment planning can be compensated using physical wedges. Enhanced dynamic wedges (EDW) were introduced by Varian to overcome some of the shortcomings of physical wedges. The objectives of this study were to measure EDW output factors for 6 MV and 20 MV photon energies for a Varian 2300CD. Secondly, to review the literature in terms of published enhanced dynamic wedge output factors (EDWOF) for different Varian models and thereby add credence to the case of the validity of reference databases. The enhanced dynamic wedge output factors were measured for the Varian 2300CD for both 6 MV and 20 MV photon energies. Twelve papers with published EDWOF for different Varian linac models were found in the literature. Comparing our results with the published mean, we found an excellent agreement for 6 MV EDWOF, with the percentage differences ranging from 0.01% to 0.57%, with a mean of 0.03%. The coefficient of variation of published EDWOF ranged from 0.17% to 0.85% and 0.1% to 0.9% for the for 6 MV and 18 MV photon energies, respectively. This paper provides the first published EDWOF for 20 MV photon energy. In addition, we have provided the first compendium of EDWOFs for different Varian linac models. The consistency of value across models and institution provide further support that a standard dataset of basic photon and electron dosimetry could be established as a guide for future commissioning, beam modeling, and quality assurance purposes.

PACS numbers: 87.50, 87.55

## I. INTRODUCTION

Physical wedges are integral part of radiation therapy treatment planning and are a well‐established method for dose inhomogeneity compensation. However, physical wedges come with well‐known problems. Firstly, physical wedges are designed for a limited field size and wedge angle. Typically, only four physical wedge angles of 15°, 30°, 45°, and 60° have been implemented by all manufacturers. Secondly, the wedge factor is dependent on many variables including beam energy, field size, depth of measurement, and type of accelerator.^(1)^ This is because of beam degradation by the physical wedge. These problems can cause dosimetric issues in treatment planning, and also any occasional misalignment of the wedge can produce significant dosimetric error in treatment delivery.^(2)^ Lastly, physical wedges are heavy and cumbersome to use clinically for the therapists (usually must be lifted overhead) and also they present a safety concern for the patient.

To resolve some of these issues with physical wedges an alternative was proposed whereby the wedge effect can be implemented by computer‐controlled movement of one of the collimator jaws (the Y jaw) and variation in output rate during treatment.^(3)^ This implementation was given a different name by each of three different manufacturers: virtual, electronic, and dynamic (Siemens, Elekta, and Varian, respectively). Descriptions of these computer‐controlled wedges have been provided in the literature.^4^, ^5^ They have the potential for any arbitrary wedge angle and fixed dimension, instead of the traditional four wedge angles with limited field sizes.

In Varian linacs, the dynamic wedge was further improved into enhanced dynamic wedge (EDW). Varian linacs use the segmented treatment table (STT), which governs the output rate (hence the delivered monitor units (MUs)) at each position of the moving jaw during treatment. All treatment STTs are generated from a single energy‐dependent STT known as the golden STT (GSTT). The GSTT is a transmission table that produces a maximum wedge angle. The concept is that an intermediate sized wedge may be produced by the linear combination of the distribution from an open field and a maximum wedge field. The maximum wedge angle is 60°, with the range of possible wedge angles varying from 10° to 60° in seven discrete angles (10°, 15°, 20°, 25°, 30°, 45°, and 60°).^(6)^ The moving jaw travels a maximum distance of 29.5 cm with 9.5 cm across the central axis. The EDW also allows the use of asymmetric fields. The fixed jaw position always corresponds to the thin end of the wedge.

The characteristics of the enhanced dynamic wedge has been studied and reported in the literature by a few authors.^6^, ^7^ Specifically, there have been reports on how to implement or commission the EDW for different treatment planning such as Pinnacle^8^, ^9^, ^10^ and Focus.^2^, ^11^ Some other studies have been centered on the EDW factors calculations.^7^, ^12^, ^13^ There are other reports that have attempted to address aspects of quality assurance of EDW.^(14)^

Linear accelerator (linac) characterization, in terms of percentage dose, output factors, and wedge factors, is critical for accurate treatment planning. In order to minimize errors, an establishment of a standard reference database for specific machine and model has been suggested.^(15)^ However, this is a very contentious topic. On one hand, there are those of the school of thought that linear accelerators cannot be manufactured to the same specifications and thus reference databases are not accurate.^(16)^ On the other hand, there are those who believe that, in the current age of computer design, linacs can be produce to very tight specifications and thus reference database are appropriate.^17^, ^18^ AAPM Task Group 106 cautions that careful evaluation is needed before using reference database in linacs' commissioning, but states that it is an excellent source of quality assurance for verifying commissioning results.^(19)^ This was a recent point/counter point debate in the *Medical Physics* journal, where it was argued whether or not reference databases or vendor‐provided databases are appropriate.^(20)^ We recently commissioned an enhanced dynamic wedge at our facility on a Varian 2300CD in Pinnacle treatment planning system version 9.2 (ADAC; Philips Healthcare, Andover, MA). In the current version of Pinnacle, the only required data are the golden STT and output factors.

The objectives of our study were, therefore, to measure EDW output factors for 6 MV and 20 MV photon energies for a Varian 2300CD. To the best of our knowledge no published data exist for the EDW output factors for Varian 20 MV photon energy. The second part of this study looks at the usefulness and accuracy of a reference database for EDW in particular. So the aim of the second part of this study was to review the literature in terms of published enhanced dynamic wedge output factor (EDWOF). Then, after generating a compendium of published EDWOF, we analyzed the EDWOF from different authors in terms of machine models, energy, and measurement conditions. This review of published EDWOF will provide further justification (or lack of) for the need of a general database.

## II. MATERIALS AND METHODS

### A. Enhanced dynamic wedge output factors (EDWOF)

A dual high‐energy (6–20 MV) linear accelerator, Varian 2300CD (Varian Medical Systems, Inc., Palo Alto, CA), installed at our Radiotherapy Department, was used to generate wedge‐shaped dose distributions by means of the enhanced dynamic wedge.

Enhanced dynamic wedge output factors (EDWOFs) of 6 and 20 MV photon beams energies were measured along the central axis using a Farmer‐type ion chamber. The ion chamber was positioned at the geometrical center of the field at a depth of 10 cm in a $40\times 40\times 40\;{\text{cm}}^{3}$ Solid Water phantom at a source‐to‐surface distance (SSD) of 100 cm. Charge was collected for a fixed number of monitor units (200 MU) for each EDW angle using PTW ion chamber (model N30002, Hicksville, NY) and electrometer Max 4000 (Sun Nuclear, Melbourne, FL). The ion chamber was oriented along the nonwedged direction. Measurements were carried out with moving jaw Y1 for both collimator rotation 0° and 180°, so as to include setup errors. This was repeated for the open field and reference $10\times 10\;{\text{cm}}^{2}$ open field using the same number of monitor units. Three measurements were acquired for each setup and then a mean value computed. Symmetric fields ranging from 5 to 20 cm were measured. It worth noting that Klein et al.^(6)^ reported that the nonwedged field dimension was found to be inconsequential for EDWOF.

The EDWOF at depth, d, in water phantom, for a field size (FS) along the central axis of the beam was calculated using Eq. (1), ^*^:

(1) $$EDWOF(\alpha ,FS,d)=D_{w}(\alpha ,FS,d)/D_{O}(FS,d)$$

where $D_{W}(\alpha ,FS,d)$ is the dose at a specified point “d” along the central axis in a specified field size “FS” with the wedge of nominal wedge angle *α* in place, and $D_{O}(FS,d)$ is the dose at the same point in an open field of equal dimensions for the same number of MU.

For Pinnacle treatment planning system (TPS) the wedge output factor is defined as the relative output factor and is calculated using Eq. (2):

(2) $$\text{Relative}\;Output\;Factor\;(ROF)=D_{w}(FS,d)/D_{C}(FSc,d)$$

where $D_{W}(FS,d)$ is the dose at a specified point “d” along the central axis in a specified field size “FS” with the wedge in place, and $D_{C}(FS,d)$ is the dose at the same point in an open calibration field for the same number of MU. In our center, the calibration field was a $10\times 10\;{\text{cm}}^{2}$.

All measurements were made at the depth of 10 cm. However, it is worth noting that depth of measurement is not critical as long as the open and wedged fields' depths are the same.^(13)^

### B. Literature search

In order to compile enhanced dynamic wedge factors, we searched PubMed database using the key word “enhanced dynamic wedge” and it resulted in 42 entries published between 1997 and 2013. Searching using the key word “dynamic wedge” resulted in 71 articles from 1989 to 2013. After reviewing all the entries we found 12 articles with published EDWOF for different Varian linac models (See Table 1).

**Table 1:** Study characteristics of the various published enhanced dynamic wedge output factors for different Varian models

	*Pasquino et al.* ^(23)^	*Shao et al.* ^(8)^	*Gibbons* ^(13)^	*Klein et al.* ^(6)^	*Koken et al.* ^(26)^	*Leavitt et al.* ^(25)^	*Ahmad et al.* ^(21)^	*Hrbacek et al.* ^(22)^	*Kuperman* ^(24)^	*Ahmad et al.* ^(29)^
Linac	DHX‐S	21EX	2100C	21N	2300	2100C	2100 C/D	2100 C/D	23 EX	2100 C
Energy	6, 15	6, 18	6, 18	6, 18	6, 15	6, 18	6, 10	6, 10	6, 18	6, 15
Ion Chamber	an IC 13	Farmer‐type ion	0.6cc PTW N23333	0.6cc PTW N23333	IC‐10 1959	ns	PTW N30002	CC13	PTW M23343	FC65–G Farmer‐type
Electrometer	ns	ns	Keithley 35614	Keithley 602	Keithley 35040	ns	ns	Keithley 35040	Keithley K602	ns
Medium	water	Solid water	Solid water	ns	ns	ns	virtual water	ns	solid water	water
Depth (cm)	10	5	dmax	10	10	ns	10	ns	10	10
SSD (cm)	90	100	ns	ns	90	ns	100	ns	100	ns

ns=not stated

### C. Data analysis

A linear regression analysis was conducted to see if field size was a predictor of our measured EDWOF. A one‐way between subjects analysis of variance (ANOVA) was conducted to compare the effect of different models and institutions on the EDWOF reported in the literature. It is worth noting that we did not have enough same models to evaluate the effect of institutions alone on the EDWOF.

To further evaluate the variance of output factor with field size across the various institutions and models, we calculated the coefficient of variation (CV). The standard formulation of the CV is the ratio of the standard deviation to the mean multiplied by 100. All these statistical analysis were carried out using Excel version 2013.

## III. RESULTS

### A. EDWOF — field size dependence

**Figure 1:**
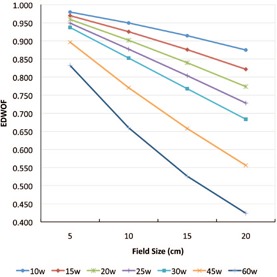
6 MV photon energy enhanced dynamic wedge output factors for Varian 2300CD as a function of field size (cm).

**Figure 2:**
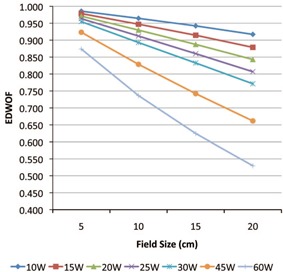
20 MV photon energy enhanced dynamic wedge output factors for Varian 2300CD as a function of field size (cm).

The EDWOF as computed using Eq. (1) as a function of field sizes are presented in Fig. 1 and 2 and in 1, 3 for 6 MV and 20 MV photon energies, respectively. The measurement precision varies from 0.1% to 0.5%. The enhanced dynamic wedge relative output factor as computed using Eq. (2) are presented in 4, 5 for 6 MV and 20 MV photon energies, respectively. The measurement precision varies from 0.1% to 0.5%. There is a linear negative dependence of EDWOF with field size (FS). When the EDWOF as a function of field size was analyzed using simple linear regression analysis, the regression coefficients varied from 0.998 to 1.00. The dependence of EDWOF on FS was also affected by the wedge angle. The highest change in EDWOF with field size is for the 60° wedge. For example, there is a 49% decrease in wedge factor between $5\times 5\;{\text{cm}}^{2}$ to $20\times 20\;{\text{cm}}^{2}$ for 60° wedge as compared to 10% for 10° wedge for 6 MV photon energy. As would be expected, the wedge factors increase with energy.

**Table 2:** Varian 2300CD enhanced dynamic wedge output factors (EDWOF) for 6 MV photon energy

*Field Size (cm)*	*Wedge Angle*						
	*10W*	*15W*	*20W*	*25W*	*30W*	*45W*	*60W*
5×5	0.980	0.970	0.959	0.949	0.937	0.896	0.832
10×10	0.949	0.925	0.902	0.877	0.853	0.771	0.660
15×15	0.914	0.876	0.840	0.804	0.768	0.658	0.526
20×20	0.875	0.822	0.774	0.728	0.684	0.556	0.424

**Table 3:** Varian 2300CD enhanced dynamic wedge output factors (EDWOF) for 20 MV photon energy

*Field Size (cm)*	*Wedge Angle*						
	*10W*	*15W*	*20W*	*25W*	*30W*	*45W*	*60W*
5×5	0.986	0.979	0.971	0.963	0.955	0.923	0.874
10×10	0.964	0.947	0.930	0.912	0.893	0.829	0.736
15×15	0.942	0.914	0.887	0.860	0.833	0.742	0.625
20×20	0.917	0.879	0.843	0.807	0.771	0.662	0.529

**Table 4:** The enhanced dynamic wedge relative output factors calculated according to Eq. (2) for 6 MV photon energy. This is the definition used in Pinnacle treatment planning

*Field Size (cm)*	*Wedge Angle*						
	*10W*	*15W*	*20W*	*25W*	*30W*	*45W*	*60W*
5×5	0.878	0.869	0.860	0.851	0.840	0.803	0.746
10×10	0.949	0.925	0.902	0.877	0.853	0.771	0.660
15×15	0.966	0.925	0.887	0.849	0.811	0.695	0.556
20×20	0.954	0.896	0.843	0.794	0.745	0.606	0.462

**Table 5:** The enhanced dynamic wedge relative output factors calculated according to Eq. (2) for 20 MV photon energy. This is the definition used in Pinnacle treatment planning

*Field Size (cm)*	*Wedge Angle*						
	*10W*	*15W*	*20W*	*25W*	*30W*	*45W*	*60W*
5×5	0.911	0.979	0.971	0.963	0.955	0.923	0.874
10×10	0.964	0.947	0.930	0.912	0.893	0.829	0.736
15×15	0.969	0.914	0.887	0.860	0.833	0.742	0.625
20×20	0.959	0.879	0.843	0.807	0.771	0.662	0.529

### B. EDWOF — comparison with published EDWOF

Our measured EDWOF compared very well with published data (see 6, 7). There were no published data for our exact model (2300CD). However, comparing our results with the published mean, we found an excellent agreement for 6 MV EDWOF with the percentage differences ranging from 0.01% to 0.57%, with a mean of 0.03%. There are no reported EDWOF for the 20 MV photon energy. However, comparing our EDWOF results (for 20 MV) with published EDWOF for 18 MV shows good agreement. The agreement ranges from 0.4% to 1.79%, with a mean of 0.55%. As expected, there is poorer agreement between the higher wedge angles for the 18 MV and 20 MV photons energies.

**Table 6:** The published EDWOF for different Varian linac models from different institutions (see Table 1 for details of the institutions) for 6 MV photon energy

*Wedge Angle*	*Field Size (cm)*	*Pasquino* ^(23)^	*Shoo* ^(8)^	*Gibbons* ^(13)^	*Klein* ^(6)^	*Koken* ^(26)^	*Leavitt* ^(25)^	*Ahmad* ^(23)^	*Hrbacek* ^(22)^	*Kuperman* ^(24)^	*Ahmad* ^(29)^	*Gibbons* ^(13)^	*current*	*mean*	*Stdev*	*CV (%)*
10	5×5	0.981	0.981	0.977	0.978		0.982					0.98	0.980	0.980	0.0018	0.18
	10×10	0.951	0.951	0.941	0.947		0.941	0.952	0.951			0.951	0.949	0.948	0.0044	0.46
	15×15	0.917	0.915	0.914	0.913		0.913					0.917	0.914	0.915	0.0017	0.18
	20×20	0.877	0.877	0.876	0.873		0.874					0.877	0.875	0.876	0.0016	0.19
15	5×5	0.972	0.971	0.968	0.967		0.967			0.972		0.97	0.970	0.970	0.0021	0.21
	10×10	0.927	0.927	0.923	0.923	0.924	0.921	0.927	0.925	0.925	0.923	0.927	0.925	0.925	0.0020	0.22
	15×15	0.879	0.88	0.879	0.873		0.874			0.874		0.879	0.876	0.877	0.0028	0.32
	20×20	0.824	0.824	0.825	0.819		0.823			0.819		0.825	0.822	0.823	0.0025	0.30
20	5×5	0.961	0.961	0.959	0.956		0.959					0.959	0.959	0.959	0.0017	0.17
	10×10	0.904	0.902	0.899	0.898		0.895	0.901	0.902			0.903	0.902	0.901	0.0028	0.31
	15×15	0.843	0.843	0.84	0.837		0.839					0.842	0.840	0.841	0.0022	0.27
	20×20	0.776	0.776	0.775	0.768		0.771					0.777	0.774	0.774	0.0032	0.42
25	5×5	0.952	0.947	0.945	0.945		0.946					0.948	0.949	0.947	0.0025	0.27
	10×10	0.88	0.88	0.873	0.873		0.872	0.877	0.88			0.88	0.877	0.877	0.0034	0.39
	15×15	0.807	0.806	0.807	0.797		0.803					0.807	0.804	0.804	0.0036	0.45
	20×20	0.73	0.729	0.733	0.725		0.727					0.731	0.728	0.729	0.0026	0.36
30	5×5	0.94	0.938	0.934	0.932		0.932			0.934		0.937	0.937	0.936	0.0029	0.31
	10×10	0.856	0.854	0.852	0.851		0.847	0.852	0.853	0.85	0.849	0.855	0.853	0.852	0.0026	0.31
	15×15	0.772	0.771	0.772	0.761		0.766			0.767		0.772	0.768	0.769	0.0039	0.51
	20×20	0.686	0.684	0.695	0.682		0.686			0.683		0.688	0.684	0.686	0.0041	0.60
45	5×5	0.901	0.897	0.896	0.891		0.892			0.893		0.895	0.896	0.895	0.0032	0.36
	10×10	0.774	0.771	0.772	0.765		0.762	0.769	0.77	0.765	0.763	0.774	0.771	0.769	0.0043	0.56
	15×15	0.661	0.662	0.662	0.653		0.654			0.652		0.663	0.658	0.658	0.0045	0.69
	20×20	0.56	0.556	0.564	0.555		0.554			0.551		0.562	0.556	0.557	0.0044	0.78
60	5×5	0.84	0.833	0.828	0.83		0.825			0.827		0.831	0.832	0.831	0.0046	0.55
	10×10	0.666	0.659	0.662	0.653	0.663	0.65	0.658	0.658	0.657	0.65	0.665	0.660	0.658	0.0053	0.81
	15×15	0.533	0.532	0.534	0.523		0.526			0.525		0.534	0.526	0.529	0.0045	0.86
	20×20	0.428	0.422	0.429	0.424	0.42	0.423			0.421		0.43	0.424	0.425	0.0036	0.85

Current=the present study; Stdev=standard deviation for the EDWOF for that field size; CV=coefficient of variation for the EDWOF for that field size.

**Table 7:** The reported EDWOF for different Varian linac models from different institutions (see Table 1 for details of the institutions) for 10 MV, 15 MV, 18 MV, and 20 MV

*Wedge Angle*	*Field Size (cm)*	*Shoo* ^(8)^ *18MV*	*Gibbons* ^(13)^ *18MV*	*Leavitt* ^(25)^ *18MV*	*Kuperman* ^(24)^ *18MV*	*Gibbons* ^(13)^ *18MV*	*Pasquino* ^(23)^ *15MV*	*Koken* ^(26)^ *15MV*	*current 20MV*	*Hrbacek* ^(22)^ *10MV*	*mean 18MV*	*Stdev 18MV*	*CV (%)*
10	5×5	0.986	0.984	0.986		0.985	0.984		0.986		0.985	0.0010	0.10
	10×10	0.964	0.965	0.958		0.964	0.961		0.964	0.957	0.963	0.0032	0.33
	15×15	0.941	0.943	0.937		0.94	0.933		0.942		0.940	0.0025	0.27
	20×20	0.913	0.914	0.917		0.914	0.905		0.917		0.915	0.0017	0.19
15	5×5	0.979	0.978	0.978	0.978	0.977	0.976		0.979		0.978	0.0007	0.07
	10×10	0.946	0.946	0.945	0.941	0.946	0.942	0.942	0.947	0.937	0.945	0.0022	0.23
	15×15	0.913	0.914	0.909	0.91	0.912	0.904		0.914		0.912	0.0021	0.23
	20×20	0.874	0.876	0.876	0.87	0.874	0.863		0.879		0.874	0.0024	0.28
20	5×5	0.971	0.973	0.969		0.969	0.968		0.971		0.971	0.0019	0.20
	10×10	0.93	0.93	0.924		0.928	0.923		0.930	0.916	0.928	0.0028	0.30
	15×15	0.885	0.886	0.881		0.884	0.874		0.887		0.884	0.0022	0.24
25	5×5	0.964	0.965	0.96		0.961	0.959		0.963		0.963	0.0024	0.25
	10×10	0.911	0.911	0.906		0.91	0.903		0.912	0.896	0.910	0.0024	0.26
	15×15	0.857	0.859	0.85		0.856	0.846		0.860		0.856	0.0039	0.45
	20×20	0.801	0.801	0.802		0.801	0.785		0.807		0.801	0.0005	0.06
30	5×5	0.955	0.957	0.95	0.951	0.952	0.949		0.955		0.953	0.0029	0.31
	10×10	0.891	0.895	0.886	0.889	0.891	0.883		0.893	0.875	0.890	0.0033	0.37
	15×15	0.829	0.824	0.822	0.824	0.828	0.815		0.833		0.825	0.0030	0.36
	20×20	0.763	0.77	0.763	0.761	0.764	0.748		0.771		0.764	0.0034	0.45
45	5×5	0.924	0.919	0.919	0.919	0.919	0.916		0.923		0.920	0.0022	0.24
	10×10	0.826	0.824	0.822	0.821	0.825	0.813		0.829	0.8	0.824	0.0021	0.25
	15×15	0.737	0.735	0.727	0.73	0.736	0.72		0.742		0.733	0.0043	0.59
	20×20	0.652	0.662	0.651	0.645	0.653	0.631		0.662		0.653	0.0061	0.94
60	5×5	0.874	0.873	0.865	0.867	0.868	0.862		0.874		0.869	0.0039	0.45
	10×10	0.732	0.73	0.725	0.726	0.732	0.715	0.723	0.736	0.698	0.729	0.0033	0.45
	15×15	0.619	0.616	0.607	0.611	0.618	0.596		0.625		0.614	0.0051	0.83
	20×20	0.521	0.522	0.52	0.515	0.523	0.498	0.497	0.529		0.520	0.0031	0.60

Current=represents the present study; Stdev=standard deviation for the EDWOF for that field size and only for the 18 MV; CV=coefficient of variation for the EDWOF for that field size and only for the 18 MV photon energy.

### C. Published EDWOF from literature review

The literature search that we conducted resulted in 12 papers with published EDWOF and the different models of Varian linacs, photon energies, measurement setup, and measurement equipment are presented in Table 1, ^6^, ^8^, ^13^, ^21^, ^22^, ^23^, ^24^, ^25^, ^26^ This includes a variety of Varian linac models and photon energies namely: 2300C/D, 21EX, 2100C, 2300, and DHX‐S, for 6 MV, 10 MV, 15 MV, and 18 MV photon energies. The published EDWOF for 6 MV photon energy for the different Varian models are presented in Table 6. The published EDWOF for 10 MV, 15 MV, 18 MV, and 20 MV are presented in Table 7. It is worth noting that Gibbons,^(13)^ Klein et al.,^(6)^ and Leavitt et al.^(25)^ reported their EDWOFs in graphical forms. The numbers presented in 6, 7 were extracted from the graphs by the current authors.

The mean EDWOF, standard deviation, and coefficient of variation (CV) are presented in the last three columns. Typically the CV for a single variable aims to describe the dispersion of the variable in a way that does not depend on the variable's measurement unit. The higher the CV, the greater the dispersion in the variable. In our case, we are looking at the dispersion of the EDWOF for the different field sizes. As you can see in Table 6, the EDWOF CV ranged from 0.17% to 0.85% for the 6 MV. Table 7 has data for 10 MV, 15 MV, 18 MV, and 20 MV. However, the mean, standard deviation and CV in Table 75 were computed for the 18 MV photon energy only. The CV ranged from 0.1% to 0.9%. This observed variation between models and institution is within experimental errors. Watts^(16)^ reported EDWOF measurement precision to be 0.2%

Analysis of variance (ANOVA) was used to evaluate the global differences in the data between the different reported EDWOF. For 6 MV photon energy EDWOF (Table 6), there was no significant difference between the different reported values (F(9,212)=0.603, p−value=0.79). Similarly there was no significant difference for reported EDWOF for 18 MV (F(2,81)=0.012, p−value=0.98). The data in 6, 7 are further confirmation that the EDWOF can be represented by a single database.

It is worth noting that Miften et al.^(11)^ and Saminathan et al.^(27)^ also published their EDWOF for Varian Clinac DHX and C‐series (unidentified model), respectively. However, their results were published in graphical format and the authors of the current paper found it difficult to extract the numerical values. Hence, the data from the Miften and Saminathan studies are not included in 6, 7. Shao et al.^(8)^ measured the EDWOF for both Varian 21EX and 2100C, but they only reported the data for 21EX.

## IV. DISCUSSION

### A. EDWOF — Dependence on field size

There was a linear depends of EDWOF with field size and this agrees with what has been reported by others in the literature.^6^, ^8^, ^21^ The dependence of EDWOF with field size is more pronounced with thicker wedge. The decrease in EDWOF with field size was explained by Ahmad et al.^(21)^ by considering the mechanism of EDW. EDW uses variable dose rate and jaw speed which contribute high doses to the toe side of the wedge field with increased field size and, consequently, central axis accumulated dose decreases, which causes a decrease in the central axis wedge factor.

### B. EDWOF — Dependence on model, energy, and institution

As presented in Table 1, the enhanced dynamic wedge output factor (EDWOF) for different Varian models have been reported in the literature by a few researchers. As evident in 6, 7, there is a good agreement across the different models and institutions (p=0.79 and p=0.98 for 6 MV and 18 MV, respectively). Klein et al.^(6)^ and Leavitt et al.^(25)^ have reported EDWOF across different Varian models. Klein and colleagues measured the EDWOF for four Varian dual‐photon energy treatment units, designated as 23N, 23S, 21N, and 21S. All four machines operated with photon energies of 6 MV and 18 MV. The machines were equipped with MLC, with 52 leaves (26 cm available) on the 23S and 21N machines and 80 leaves (40 cm) on the 21S and 23N. The Klein study found that the measured EDWOF for all four machines were within ±1.5%. Similarly Leavitt and colleagues carried out independent measurements of EDWOF at four different institutions and five different linear accelerators, and showed very good inter‐institutional agreement. Our results further confirm the findings of the Klein and Leavitt studies of nonsignificant variation in EDWOF across models and across institutions.

These results are not surprising since the principle of photon generation by the Varian linac is the same across the different models. The difference across models is mostly due to add‐ons like MLC, cone‐beam CT, and number of electron beams energies. The nominal accelerating potential that determines the quality of the beam and hence affects the EDWOF remains very similar across the different models, and hence the observed similarity in EDWOF across models. For example, Watts^(16)^ measured the mean PDD for six Varian 2100C models at the depth of 10 cm×10 cm field size to be 66.7% and standard deviation of 0.4%

As seen in Table 1, the measurement setup was not the same across the different publications. The depth of measurement and the SSD varied between centers. However, the depth of measurement has been reported to be inconsequential on EDWOF measurement. Avadhani et al.,^(28)^ Ahmad et al.,^(29)^ Gibbons,^(13)^ and Ahmad et al.^(21)^ demonstrated that, unlike physical wedges, EDWOF were independent of depth. Ahmad and colleagues observed a less than 2% variation in EDWOF with depth. Gibbons measured wedge factors at four different depths (1.5, 5, 7.5, and 10 cm) for three Clinac 2100C and two Clinac 600CD accelerators and found the EDWOF to be relatively insensitive to measurement depth. While Ahmad and colleagues observed a nonsignificant variation of EDWOF with depth, they found up to 8.9% variation in 60° physical wedge with depth for a 6 MV photon. This lack of depth dependence for dynamic wedge can be explained by the absence of beam hardening.

### C. General reference data

The second part of this paper looks at reference database, the validity of which has recently been debated in the literature.^(20)^ One of the reasons for the support of reference database is that it has the potential to provide a well‐controlled and collected data with probably the least error. This is because the process of commissioning a linac requires, among other tasks, the acquisition and processing of a significantly large amount of machine beam data.^(19)^ The number of measurements involved is so large that the entire process is considered to be one of the most complex and error‐prone in radiation oncology today.^(30)^ The need for high accuracy in data collection can never be overstated. In most cases, beam data collected during commissioning a linac are treated as reference and performed only once in the lifetime of the machine. A credentialing study conducted by the Radiological Physics Center (RPC) found an alarming number of institutions (30%) failing to pass clinically acceptable tolerance limits of 7% dose or 4 mm distance to agreement in their phantom irradiations. Incorrect output factors and percentage depth doses were identified as some of the causes of failure.^(31)^ Several recent reports of radiation incidents have been associated with commissioning errors.^(32)^

The construction and dosimetric characteristics of modern computer‐controlled linear accelerators are generally very reproducible, and this has been attributed to improved machining techniques during manufacturing and the use of computers during operation.^(16)^ This has been supported by studies that demonstrated the equivalence of either locally collected data to vendor‐provided beam data, or data for machines of the same models.^15^, ^16^, ^18^, ^33^ Cho et al.^(18)^ from the RPC, for example, measured data on over 50 Varian Clinac 2100 units and found that all dosimetric data were within the clinically acceptable range of less than 1%. They also found acceptable agreement with the vendor machine data. Sjostrom et al.^(34)^ studied the characteristics of eight Varian iX accelerators with 6 and 15 MV photon beams and 6–18 MeV electrons and concluded that all photon and electron beams, except the 15 MV photon beam from two accelerators, could be represented by one set of data. Recent comparisons among models and across institutions have been reported for the newer Varian models, TrueBeam.^35^, ^36^, ^37^ Beyer^(35)^ found that the PDD and profile data between the TrueBeam, Clinac, and Trilogy linear accelerator to be almost identical, with less than 1% variation. Glide‐Hurst et al.^(36)^ compared data from five Varian TrueBeam linacs at three institutions and found good agreement between machines for dosimetric data. For example, the photon and electron PDDs were comparable for all energies, with a variation of less than 0.3%. An interesting observation is that there was a high agreement between machines despite the fact that the machines were not “matched”. (Beam matching is when the linear accelerators are set within the manufacturer's specifications or set to a specific dataset within the manufacturer specification range.^(34)^) These recent comparative studies provide no information on wedge factors (physical or dynamic). However, the similarity of data presented in 6, 7 amongst different institutions and models further support the accuracy of using reference data.

It is worth noting that this concept of reference data is not limited only to Varian linacs.^17^, ^38^ Song et al.^(38)^ analyzed the 6 MV photon beams for seven Philips/Elekta linacs of five different models: SL 75/5, SL 15, SL 25, SLi Precise, and SLi. They observed a highly consistent beam quality across models as defined by %dd(10)χ of 67.7% (±0.3%). Cho and Ibbott^(17)^ from the Radiological Physics center studied the dosimetric characteristics of Siemens Primus at eight different institutions. They found that the RPC‐measured output factors for the Primus varied by less than 2% for each field size across all eight centers.

From the above discussions it can therefore be argued that the use of general reference database has potential advantages in radiation therapy, namely:

1. It can provide quick reference for clinical medical physicists to compare their field machine characteristics to established databases.
2. It can provide quality assurance for machine characteristics.
3. Such data can be imported into MU double‐check programs to provide true double check of treatment planning MUs. Current practice of importing data from treatment planning program is not truly optimal. This is because if an error occurred during commissioning, for example a wrong field size, such error will also be propagated into the double‐check program. Hence, the double‐check program needs an independent set of data to provide true double‐check analysis.
4. It can be used as the standard data to be imported into treatment planning systems (TPS). If such reference data are used in the TPS, caution should be exercised and the in‐house physicist should do an extensive validation of the data.

With the forgone justification and discussions, it is worth encouraging either researchers to publish their commissioning data or manufacturers of linacs to create a repertoire for data collection.

### D. Clinical use

Finally, it is important for the clinical physicist to commission dynamic wedges (or virtual wedges) because they have been shown to have significant benefits for both the therapists and the patients. When compared with physical wedges, these nonphysical wedges have several clinically relevant advantages, including reduction of treatment time, less scatter dose to peripheral areas and, in many cases, extended field size capabilities. There is significant dose reduction in areas outside the treatment field by using the dynamic wedge instead of the physical wedge.

For breast treatment the enhanced dynamic wedge has practical clinical advantage. There is an improved dose distribution in patients undergoing breast conservation therapy while at the same time minimizing dose to the contralateral breast, thereby reducing the potential carcinogenic effects. The study by Warlick et al.^(39)^ revealed a significant reduction in the contralateral breast dose with the enhanced dynamic wedge compared to the standard metal wedge. The dose was measured at varying distances from the geometric field edge, ranging from 2 to 8 cm. The average dose with the enhanced dynamic wedge was 2.7%–2.8%. The average dose with the standard wedge was 4.0%–4.7%. Thermoluminescent dosimeter measurements suggest an increase in both scattered electrons and photons with metal wedges.

Enhanced dynamic wedges have the disadvantage of requiring more frequent QA on its functionality. These requirements are listed in AAPM Task Group report number 142 (see Table IV).^(40)^

### E. Weaknesses of the current study

1. There are no published enhanced dynamic wedge output factors for newer Varian models such as the Trilogy and TrueBeam.
2. This study was only limited to the Varian linacs.

## V. CONCLUSIONS

We reported the enhanced dynamic wedge output factors for both 6 MV and 20 MV photon energies for Varian 2300CD. To best of our knowledge our EDWOFs are the only reported for 20 MV photon energy. In addition, we have provided first compendium of enhanced dynamic wedge output factors for different Varian linac models. The consistency of value across models and institutions provide further support that a standard dataset of basic photon and electron dosimetry could be established as a guide for future commissioning, beam modeling, and quality assurance purposes.
